# Role of Body Mass Index, Waist-to-Height and Waist-to-Hip Ratio in Prediction of Nonalcoholic Fatty Liver Disease

**DOI:** 10.1155/2012/362147

**Published:** 2012-05-31

**Authors:** Rui-Dan Zheng, Zhuo-Ran Chen, Jian-Neng Chen, Yan-Hui Lu, Jie Chen

**Affiliations:** Research and Therapy Center for Liver Disease, The Affiliated Dongnan Hospital of Xiamen University, Fujian, Zhangzhou 363000, China

## Abstract

*Objective.* To investigate the anthropometric indicators that can effectively predict the nonalcoholic fatty liver disease (NAFLD). *Methods.* The height, body weight, waist and hip circumference were measured, and body mass index (BMI), waist-to-height (WHtR) and waist-to-hip ratio (WHR) were calculated. M-H chi square test, logistic regression analysis, and receiver-operating characteristic (ROC) curve were employed for the analysis of risk factors. *Patients or Materials.* 490 patients were recruited, of whom 250 were diagnosed as NAFLD and 240 as non-NAFLD (control group). *Results.* Compared with the control group, the BMI, WHR, and WHtR were significantly higher in patients with NAFLD. Logistic regression analysis showed that BMI and WHR were effective prognostic factors of NAFLD. In addition, WHR plays a more important role in prediction of NAFLD by the area under curve. *Conclusion.* WHR is closely related to the occurrence of NAFLD. We assume that WHR is beneficial for the diagnosis NAFLD.

## 1. Introduction

Nonalcoholic fatty liver disease (NAFLD) is a cause of fatty liver occurring when fat is deposited (steatosis) in the liver, whereas not due to excessive alcohol use or other definite injuries to the liver. NAFLD is a clinical syndrome and pathologically characterized by diffuse macrovesicular fatty change in the hepatocytes. NAFLD includes simple nonalcoholic fatty liver disease, nonalcoholic steatohepatitis and hepatic cirrhosis [1]. NAFLD is not static, and the NAFLD in the childhood may develop into hepatic cirrhosis over age, which significantly results in the increase of liver disease-related mortality [2]. Early diagnosis and prompt treatment for NAFLD are helpful to block or even reverse the development of hepatic cirrhosis [3]. Liver biopsy has been a gold standard in the clinical diagnosis of NAFLD, but it is invasive and has sampling error and bias between observers which significantly limit the wide application of liver biopsy [4]. In the present study, patients with pathologically and clinically proven NAFLD were recruited, and the four anthropometric indicators including waist circumference (WC), waist-to-hip ratio (WHR), waist-to-height ratio (WHtR), and body mass index (BMI) were determined. Our study aimed to find out effective anthropometric indicators for the prediction of NAFLD.

## 2. Patients and Methods

### 2.1. Ethics Statement

Our work was conducted in accordance with the Declaration of Helsinki (1964). It was approved by the Ethical Committee of Xiamen University (XM2009-0003) and conformed to the National Institute of Health guidelines on the clinical studies. Informed consent was obtained from all patients.

### 2.2. Patients

A total of 250 patients with clinically and pathologically proven NAFLD were recruited from the Center for Liver Diseases of our hospital from 1 January 2008 to 29 June 2011. The NAFLD was diagnosed according to the criteria for NAFLD [5], developed by the Society of Hepatology of Chinese Medical Association [6]. There were 161 males and 89 females with a mean age of 36.60 ± 11.14 years (range: 15~56 years). In addition, 240 patients with non-NAFLD were also recruited in the same period and served as controls. There were 96 patients with drug-induced liver disease, 86 with toxic hepatitis, 19 with hepatic hemangioma, and 39 with autoimmune liver disease. In the control group, there were 192 males and 48 females with a mean age of 37.32 ± 10.19 years (range: 19~58 years) (Table 1). Exclusion criteria included coinfection with HBV, HIV or HCV, alcohol consumption 30 g/day. All patients were defined as persons who had negative hepatitis B surface antigen (HBsAg) for at least 6 months before enrolling.

### 2.3. Detection of Anthropometric Indicators

Measurement was done by the same experienced physicians who received specialized training. The height, body weight, waist circumference (WC), and hip circumference (HC) were measured. Patients stood without wearing shoes but wearing ordinary clothes. The WHR, WHtR, and BMI were calculated. BMI = body weight/height^2^, WHR = WC/HC, WHtR = WC/height. The WC and HC were measured at the level midway between the lowest rib and the iliac crest and at the level of the great trochanter.

### 2.4. Diagnostic Criteria: Criteria for Obesity

According to the classification of adult body weight by BMI in Asian populations, 18.5 kg/m^2^ ≤ BMI < 23.0 kg/m^2^ was defined as normal, 23.0 kg/m^2^ ≤ BMI < 25.0 kg/m^2^ as overweight, and BMI ≥ 25.0 kg/m^2^ as obesity (peripheral obesity). WHR of ≥0.9 in man and ≥0.8 in woman was defined as central obesity (+). WHtR of *⩾*0.5 was defined as obesity. 

### 2.5. Collection of Liver Samples

All patients received liver biopsy under ultrasonography within 1 week after admission. Vacuum aspiration of liver tissues was done within 1 second by using 14G Quick-cut needle (HAKKO Co., Ltd, Japan) or Menghini needle. The liver tissues were 2 cm in length, and periportal areas were more than 10. The aspirated liver tissues were fixed in 10% neutral formaldehyde followed by dehydration, embedding, and sectioning [7]. Hematoxylin-eosin (HE) staining, Masson staining, and reticular fiber staining were performed. Sections were analyzed by two pathologists who were blind to the study. The diagnosis of NAFLD was based on the criteria for NAFLD developed by the Panel of Fatty Liver and Alcoholic Liver Disease of Hepatology Branch of the Chinese Medical Association [6]. 

### 2.6. Statistical Analysis

Statistical analysis was performed with SPSS version 13.0. Quantitative data were compared with *t* test and qualitative data compared with chi square test. In the present case-control study, risk factors were categorized and the odds ratio (OR) and 95% confidence interval (CI) were calculated followed by statistical analysis with *Mantel-Haenszel *χ*^2^* test. For the multilevel BMI, dose-reaction relation was employed for analysis of risk factors. Bivariate logistic regression analysis was performed to investigate the correlation between factors and NAFLD in which the BMI, WHR, and WHtR were converted into categorical variables. Partial maximum likelihood estimation (forward selection method) was used for variable screening, and model testing was done with likelihood ratio test. Single regression coefficient was tested with *Wald* test. ROC curve was delineated with software. A value of *P* < 0.05 was considered statistically significant. 

## 3. Results

### 3.1. General Characteristics

Among these 490 patients, there were 250 patients with NAFLD and 240 with non-NAFLD. The mean age was 37.32 ± 10.19 years in NAFLD patients and 36.60 ± 11.14 years in non-NAFLD patients showing no significant difference (*F* = 0.69, *P* = 0.41; *t *= −0.49, *P* = 0.62). In the NAFLD, there were 189 males and 61 females. In the non-NAFLD group, there were 192 males and 48 females. There was no marked difference in the gender between two groups (**χ*^2^* = 1.12, *P* = 0.29). In the NAFLD group, the (*t* = 8.34, *P* < 0.001), WHR (*t*′ = 10.42, *P* < 0.001) and WHtR (*t*′ = 9.10, *P* < 0.001) were dramatically higher than those in the non-NAFLD group (Table 1). 

### 3.2. Correlation between BMI, WHR and WHtR and NAFLD

BMI, WHR and WHtR were the risk factors of NAFLD (Tables 2, 3, 4). When the BMI was ≥23.0 but <25.0, the OR was 21.83 (95% CI: 5.24~90.79; *χ*_MH_^2^ = 20.53; *P* < 0.001). When the BMI ≥ 25.0, the OR was 29.92 (95% CI: 14.54~222.15; *χ*_MH_^2^ = 56.83; *P* < 0.001). Furthermore, the OR increased with the elevation of BMI (**χ*^2^* = 25.03, *P* < 0.001). The OR of WHR was 30.52 (95% CI: 11.466~81.39; *χ*_MH_^2^ = 56.79; *P* < 0.001). The OR of WHtR was 21.04 (95% CI: 7.63~57.98; *χ*_MH_^2^ = 42.69; *P* < 0.001) (Table 5). 

### 3.3. Bivariate Logistic Regression Analysis

NAFLD was defined as the endpoint. At the same time the criteria such as age, gender, height, body weight, WC, HC, the standardized BMI, WHR, and WHtR were applied for logistic regression analysis (Table 6). Partial maximum likelihood estimation with forward selection method was used for the screening of variables. Results showed BMI $\left(\text{O}{\hat{\text{R}}}_{j}=11.76\right)$ and WHR $\left(\text{O}{\hat{\text{R}}}_{j}=3.09\right)$ were two effective indicators for the prediction of NAFLD, and the probability equation was *P *= e^−2.239 + 1.130BMI + 2.464WHR^/(1 + e^−2.239 + 1.130BMI + 2.464WHR^), (**χ*^2^* = 74.82, *P* < 0.001). 

### 3.4. ROC Curve Analysis of NAFLD-Related Indicators

**Figure 1:**
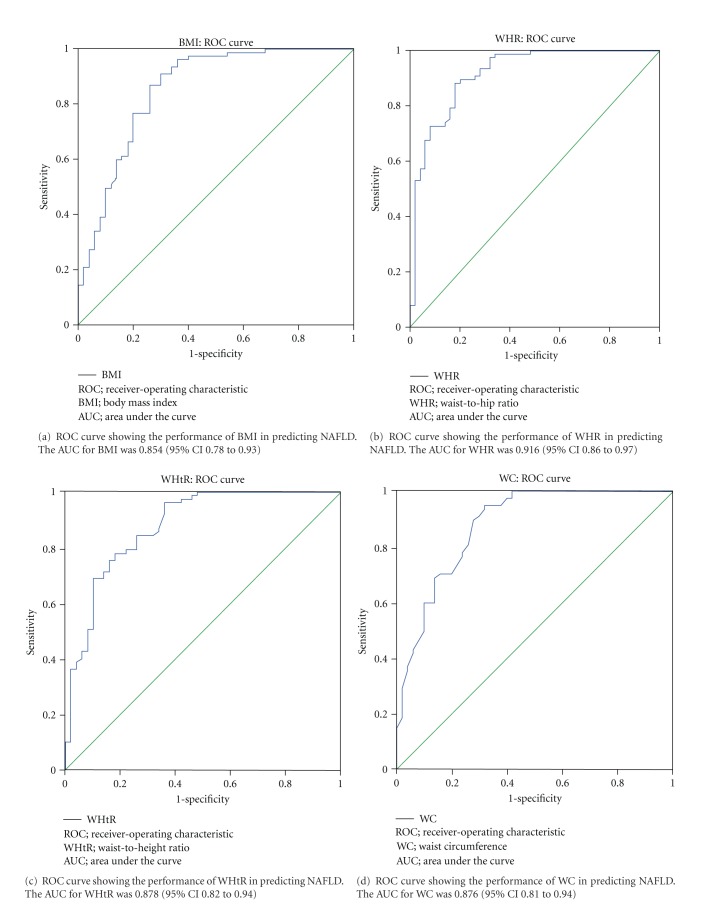
ROC curve analysis of NAFLD-related indicators. (a) BMI; (b) WHR; (c) WHtR; (d) WC.

BMI (AUC = 0.854, Figure 1(a)), WHR (AUC = 0.916, Figure 1(b)), WHtR (AUC = 0.878, Figure 1(c)), and WC (AUC = 0.876, Figure 1(d)) had diagnostic value for NAFLD and the WHR had the largest AUC and the highest diagnostic value. When the cutoff value was 0.891, the Youden index (sum of sensitivity [0.987] and specificity [0.660]) was the largest (Table 7). 

## 4. Discussion

In recent years, BMI, WHR, and abdominal circumference are changed significantly with the elevation of living standard and changes in the lifestyle, and the mean BMI in adults is increasing in both developed and developing countries. Currently, there are about 1.46 billion and 0.5 billion subjects with overweight and obese, respectively [8]. In addition, in USA, overweight people account for 60% of adults and obesity subjects for 25% of adults, and overweight and obesity have been important heath problems in children and adolescents [9]. The incidence of NAFLD is increasing, and NAFLD has become a common chronic liver disease and a critical problem threatening the human health. NAFLD has been confirmed to be closely related to the hyperlipidemia, hypertension, type 2 diabetes, cardiovascular diseases, and some malignancies [10]. In China, the NAFLD has become the second leading liver disease following viral hepatitis, which is similar to the condition in western countries [11]. The incidence of NAFLD is as high as 60%~90% in obese subjects, and the prevalence of NASH and hepatic cirrhosis was 20%~25% and 2%~8%, respectively [12]. In NAFLD patients, the incidence of type 2 diabetes is significantly increased, and the NAFLD is closely associated with malignancies and can promote the occurrence and development of atherosclerotic cardiovascular diseases [13]. Recently, more attention has been paid to the prevention and treatment of overweight and obesity in clinical practice. In addition, there is evidence showing that insulin resistance is closely related to the NAFLD and has been a hot topic in researches on NAFLD. Clinically, to determine the insulin resistance is critical, but detection of insulin level is invasive, and there is still no reference range for insulin resistance. These significantly limit the wide application of detection of insulin resistance. Liver biopsy has become a gold standard in the diagnosis of NAFLD, but it is invasive. Currently, abdominal color Doppler ultrasonography is used for the noninvasive diagnosis of NAFLD, and this technique still has limitations due to the low consistence with pathological findings. Thus, it is imperative to develop simple and sensitive indicators for the diagnosis of NAFLD. In the present study, WHR was used as an indicator of central obesity and the effect of WHR on the NAFLD was investigated. To better predict the NAFLD in high-risk population, the pathological findings were used as the main standard for the diagnosis of NAFLD, and the anthropometric indicators were also measured. 

In the present study, WC, WHR, WHtR, and BMI were measured, and statistical analysis showed WHR had advantages in the prediction of NAFLD over WC, WHtR, and BMI. In addition, logistic analysis and ROC curve analysis also revealed WHR had the largest OR (11.757), suggesting WHR is a critical anthropometric indicator for the prediction of NAFLD. However, WC and WHtR were not included into the equation. In the analysis of NAFLD with the ROC curve, WHR had the largest AUC (0.916) and thus had the best diagnostic value. When the cutoff of WHR was 0.89, the sensitivity and specificity were 0.99 and 0.66, respectively. NAFLD is closely related to the metabolic syndrome. Studies have shown that WHtR and WC are the main risk factors used for the evaluation of metabolic syndrome and coronary heart disease [14, 15]. A study on the correlation between WHR and cardiovascular events revealed WHR was a risk factor that could increase the risk for cardiovascular events [16]. Investigators have indicated WHR is a good anthropometric indicator for the prediction of type 2 diabetes [17]. Diabetes is associated with cardiovascular and cerebrovascular events. In a prospective study on diabetes revealed WHR was an important indicator that could predict the risk for cardiovascular events in type 2 diabetes patients [18]. Our results show WHR is a risk factor of NAFLD which indicates central obesity is closely related to the occurrence of NAFLD. WHR is the most important anthropometric indicator for the prediction of NAFLD and independent of other risk factors, which is consistent with previously reported [19]. Moreover, our findings also reveal BMI also has value in the prediction of NAFLD, and BMI, WHR, and WHtR are closely associated with the occurrence of NAFLD (Tables 2, 3, and 4). In the study of Aekplakorn et al. [20], results showed WC, WHR, and WHtR had minor advantages in the prediction of cardiovascular events over BMI in Thai population. However, in the Caucasian of USA and Europe, BMI has advantage in the prediction of type 2 diabetes over other indicators [21]. In addition, the ethnic difference may also affect the selection of indicators for the prediction of NAFLD. Furthermore, there is evidence showing that the prediction of cardiovascular events with WHR depends on the gender [21]. Studies on the genetic and environmental factors revealed the morbidity and outcome of NAFLD varied from regions and races [22]. In the present study, although logistic regression was used to adjust the confounding factors including gender, our findings had the tendency to predict NAFLD in males due to the high proportion of males. Our findings may be more useful in the prediction of NAFLD in males. WHR is simple to measure and thus can be applied as an important anthropometric indicator to screen population with high risk for NAFLD which is beneficial for the diagnosis and treatment of NAFLD. 

## Figures and Tables

**Table 1:** Gender, age and anthropometric indicators in NAFLD group and non-NAFLD group $\left(\overset{̅}{x}\pm s\right)$.

Clinical indicators	Non-NAFLD group (*n* = 240)	NAFLD group (*n* = 250)	*P*
Age (yr)	37.32 ± 10.19	36.60 ± 11.14	0.62
Gender (male)	192 (80%)	189 (75.6%)	0.29
BMI	24.78 ± 10.14	36.79 ± 10.12	*P* < 0.001
WHR	0.82 ± 0.03	0.98 ± 0.04	*P* < 0.001
WHtR	0.44 ± 0.08	0.59 ± 0.06	*P* < 0.001

**Table 2:** Correlation between BMI and NAFLD.

BMI (Kg/m^2^)	BMI < 23.0	23.0 ≤ BMI < 25.0	BMI ≥ 25.0	Total
X_k_	0	1	2	
NAFLD group a_k_(*T_k_)	10 (20.61)	62 (16.98)	178 (44.87)	250
Non-NAFLD group b_k_	149 (13.39)	43 (11.02)	48 (39.41)	240

Total	34	28	65	127

$\text{O}\hat{\text{R}}$	1.0	21.83	29.92	
*χ*_MH_^2^	—	20.53	56.83	
95% CI	—	5.24~90.79	14.54~222.15	

*T_k_ refers to the theoretical frequency corresponding to a_k_.

**Table 3:** Correlation between WHR and NAFLD.

WHR group	WHR ≥ 0.9 (man); WHR ≥ 0.85 (woman)	WHR < 0.9 (man); WHR < 0.85 (woman)	Total
NAFLD group	189	61	250
Non-NAFLD group	43	197	240

Total	76	50	127

$\text{O}\hat{\text{R}}$	30.52		
*χ*_MH_^2^	56.79		
*P*	4.84*E* − 14		
95% CI	11.45~81.39		

**Table 4:** Correlation between WHtR and NAFLD.

WHtR group	WHtR ≥ 0.5	WHtR < 0.5	Total
NAFLD group	185	65	250
Non-NAFLD group	86	154	240

Total	89	38	127

$\text{O}\hat{\text{R}}$	21.04		
*χ*_MH_^2^	42.69		
*P*	6.40*E* − 11		
95% CI	7.63~57.98		

**Table 5:** Risk factors and standardization of risk factors.

Risk factors	Standardization	
BMI	BMI of < 23 as 0	BMI of < 25 and ≥23 as 1; BMI of ≥ 25 as 2
WHR	WHR of < 0.9 (man); WHR of < 0.85 (women) as 0	WHR ≥ 0.9 (man); WHR ≥ 0.85 (woman) as 1
WHtR	WHtR of < 0.5 as 0	WHtR ≥ 0.5 as 1

**Table 6:** Logistic analysis of risk factors of NAFLD.

	Partial regression coefficient *B *	Standard error	*Wald *χ*^2^*	*P*	$\text{O}\hat{\text{R}}$	95% CI
WHR	2.464	0.563	19.148	1.210*E* − 3	11.757	3.899~35.454
BMI	1.130	0.355	10.127	0.001	3.094	1.543~6.204
Constant	−2.239	0.496	20.373	6.374*E* − 6	0.107	
Cox and Snell *R* ^2^	0.445					
Nagelkerke *R* ^2^	0.603					
Discriminant effectiveness	85%					

**Table 7:** Sensitivity, specificity and AUC of cutoff value of anthropometric indicators in prediction of NAFLD.

Anthropometric indicators	Cutoff value	Youden index	Sensitivity	Specificity	AUC	95% CI	*P*
BMI	24.22	0.610	0.96	0.640	0.854	0.78~0.93	1.867*E* − 3
WHR	0.89	0.703	0.99	0.660	0.916	0.86~0.97	2.621*E* − 3
WHtR	0.49	0.601	0.96	0.640	0.878	0.82~0.94	7.233*E* − 3
WC	82.50	0.628	0.95	0.680	0.876	0.81~0.94	9.139*E* − 3
